# Innate Immunity and Biomaterials at the Nexus: Friends or Foes 

**DOI:** 10.1155/2015/342304

**Published:** 2015-07-12

**Authors:** Susan N. Christo, Kerrilyn R. Diener, Akash Bachhuka, Krasimir Vasilev, John D. Hayball

**Affiliations:** ^1^Experimental Therapeutics Laboratory, Sansom Institute and Hanson Institute, School of Pharmacy and Medical Science, University of South Australia, Adelaide, SA 5000, Australia; ^2^Robinson Research Institute, School of Paediatrics and Reproductive Health, University of Adelaide, Adelaide, SA 5005, Australia; ^3^Mawson Institute, University of South Australia, Adelaide, SA 5095, Australia; ^4^School of Medicine, University of Adelaide, Adelaide, SA 5005, Australia

## Abstract

Biomaterial implants are an established part of medical practice, encompassing a broad range of devices that widely differ in function and structural composition. However, one common property amongst biomaterials is the induction of the foreign body response: an acute sterile inflammatory reaction which overlaps with tissue vascularisation and remodelling and ultimately fibrotic encapsulation of the biomaterial to prevent further interaction with host tissue. Severity and clinical manifestation of the biomaterial-induced foreign body response are different for each biomaterial, with cases of incompatibility often associated with loss of function. However, unravelling the mechanisms that progress to the formation of the fibrotic capsule highlights the tightly intertwined nature of immunological responses to a seemingly noncanonical “antigen.” In this review, we detail the pathways associated with the foreign body response and describe possible mechanisms of immune involvement that can be targeted. We also discuss methods of modulating the immune response by altering the physiochemical surface properties of the biomaterial prior to implantation. Developments in these areas are reliant on reproducible and effective animal models and may allow a “combined” immunomodulatory approach of adapting surface properties of biomaterials, as well as treating key immune pathways to ultimately reduce the negative consequences of biomaterial implantation.

## 1. Introduction

The role of implantable biomaterials is to replace or enhance biological function or offer structural support in host tissue [1, 2]. Biomaterials can be used for different lengths of time within the host depending on the intended purpose. Temporary biomaterials include contraceptive implants [3–5], pins for bone reconstruction and bone lengthening [6, 7], certain dental implants prior to restoration [8], catheters [9], stents [10], and some dermal fillers used for cosmetics and facial reconstruction [11, 12]. However, the majority of implantable biomaterials are intended for the duration of the host's lifetime. These biomaterials encompass a range of applications in many tissue types but are most prominent in and for orthopaedics [13, 14], dental settings [15, 16], cardiac pacemakers [17, 18], and glucose biosensors [19–21]. Unfortunately, invasive implantation methods, such as surgery, potentiate an unavoidable adverse host response [22] in which the biomaterial itself determines the duration of the inflammatory response. These negative consequences may contribute to the ultimate failure of the device, reducing the lifespan of the biomaterial and necessitating additional implantations [23–25]. Therefore, the longevity of the biomaterial is central to the aims of manufacturing and design whilst upholding maximum biological functionality. The aim of biomaterial research is to develop biocompatible devices that can be integrated into tissue and perform their intended functionality with minimal damage or negative response to the host [1].

## 2. The Foreign Body Response to Implantable Biomaterials

**Figure 1:**
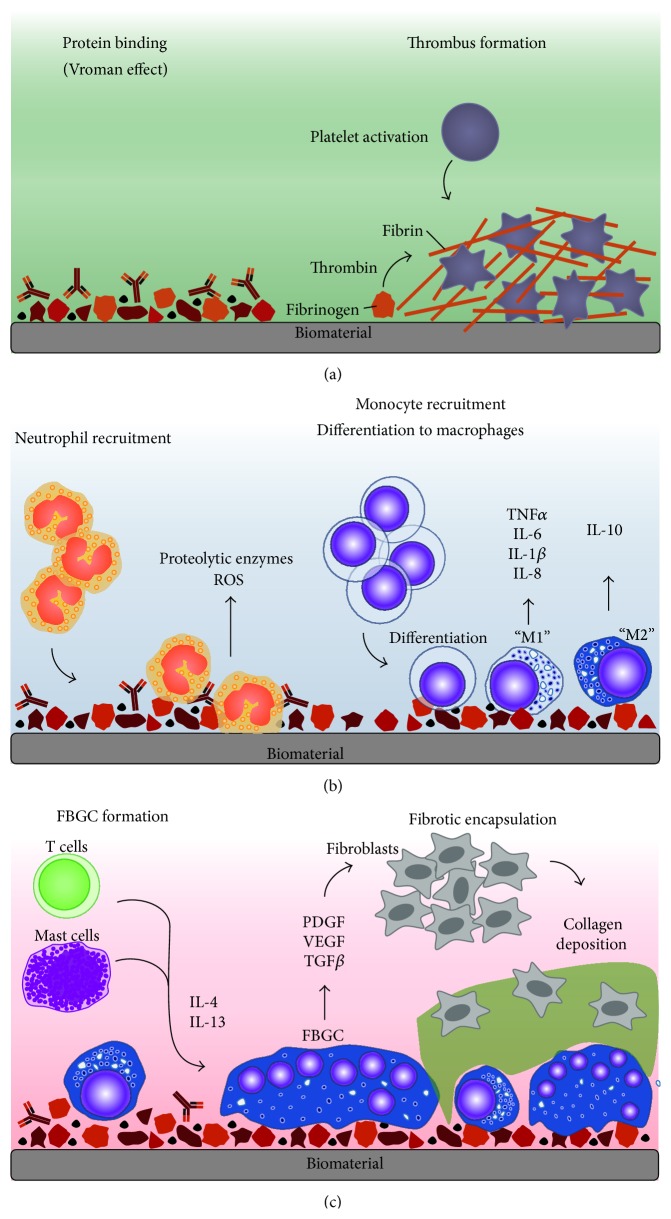
Foreign body response to biomaterials. The foreign body response is an acute inflammatory reaction which overlaps with tissue vascularisation and remodelling and ultimately fibrotic encapsulation. (a) Upon implantation, blood proteins are immediately adsorbed and instigate the formation of a thrombus, which acts as a provisional matrix rich in factors that recruit innate leukocytes. (b) Neutrophils are recruited to the site of implantation and attempt to degrade the biomaterial. Monocytes are also recruited and mature into macrophages, which undergo differentiation from an M1 and M2 phenotype and ultimately exhaust their phagocytic capacity. (c) Adaptive leukocytes, such as T cells and mast cells, are recruited and secrete cytokines that encourage foreign body giant cell (FBGC) formation. Fibroblast recruiting factors are secreted by FBGCs and result in their activation and collagen deposition, ultimately forming a capsule around the biomaterial to prevent further interaction with the host tissue.

The utility and function of biomaterial implants can be compromised by the development of a foreign body reaction (FBR): an acute sterile innate immune inflammatory reaction which overlaps with tissue vascularisation and remodelling and ultimately fibrotic encapsulation to prevent further interaction with the host tissue (Figure 1) [26–28]. All biomaterial implants have the capacity to induce a FBR; however the severity and clinical manifestation of these responses can widely differ [29]. Here, the pathways involved in the FBR are summarised, and the reader is directed to more detailed accounts of protein and cellular responses reviewed elsewhere [22, 30].

### 2.1. Protein Adsorption onto the Biomaterial Surface

The FBR is initiated within seconds of implantation [31], characterised by rapid and spontaneous adsorption and deadsorption of host serum proteins (the Vroman effect [32]) including albumin, fibrinogen, fibronectin, vitronectin, gamma globulin, complement, and other immunomodulatory proteins (Figure 1(a)) [33–37]. The resulting thrombus formation defines the provisional matrix around the biomaterial [38–41], leading to the aggregation of activated platelets and fibrin, which is formed upon thrombin-mediated conversion of fibrinogen [42]. Fibrinogen has also been shown to activate platelets [43, 44], and both fibrin and fibrinogen can recruit additional immune cells to the site of the biomaterial [45, 46]. Biomaterial-adsorbed vitronectin can enhance cell adhesion [47–49], whereas an additional adhesion protein, fibronectin, is suggested to partake in the chronic phase of the FBR [50, 51]. Importantly, this highlights how the provisional matrix is a milieu rich in activating and immunomodulatory molecules that direct acute inflammatory mechanisms [30].

### 2.2. Acute Inflammation in the FBR Cascade

Central to acute inflammation are the recruitment and activation of neutrophils (Figure 1(b)), which rapidly localise to the implantation site upon release of chemoattractants by activated platelets [52, 53] and endothelial cells [54–57]. Neutrophils attempt to destroy the biomaterial by mechanisms intrinsic to their function, which include phagocytosis and degranulation for the release of proteolytic enzymes and reactive oxygen species (ROS) [58–63]. More recently, neutrophils have been shown to release neutrophil extracellular traps (NETs) [64], comprised of a “network” of granular proteins, neutrophil elastase, chromatin DNA, and histones [65]. The “sticky” nature of these NETs is used to “trap” pathogens and prevent spread of infection, as well as possessing bactericidal activity [64]. However, the involvement of NETs, if any, has not been detailed for implantable biomaterial-induced inflammation.

In parallel to neutrophil recruitment, circulating monocytes can respond to platelet-derived chemoattractants localised to the implantation site [30] and bind the protein layer on the biomaterial via fibrinogen, resulting in their activation (Figure 1(b)) [51, 66, 67]. At the site of injury, these monocytes differentiate into classically activated or “M1” macrophages [68], defined by their ability to secrete proinflammatory cytokines, including interleukin (IL)-1*β*, IL-6, IL-8 and tumour necrosis factor (TNF)*α*, and chemokines [68, 69]. Adherent macrophages also attempt to degrade the biomaterial by releasing ROS and degradative enzymes [70, 71], before undergoing “frustrated” phagocytosis because the biomaterial is too large to internalise, ultimately resulting in an exerted increase in proinflammatory cytokines [72, 73]. Analogous to wound healing events [74], biomaterial-adherent macrophages eventually transition into an alternatively activated or “M2” phenotype [75], characterised by reduced degradative capacity, secretion of anti-inflammatory cytokines, such as IL-10, and gained tissue remodelling functionality. The overlapping events of the phenotypic M1 to M2 switch as well as the mechanisms of frustrated phagocytosis results in the fusion of macrophages into a foreign body giant cell (FBGC) on the biomaterial surface in an attempt to increase their phagocytic functionality [71, 76].

### 2.3. Chronic Inflammation in the FBR Results in Reduced Biomaterial Function

The formation of FBGCs is often a signature component of biomaterial-induced FBR and is instigated through the activation of mast cells, basophils, and T helper (Th) cells that secrete IL-4 and IL-13 secretion known to induce macrophage fusion (Figure 1(c)) [77–79]. Mast cells are consistently reported at the site of implantation [80–82] and have demonstrated activation-induced degranulation and the secretion of pro- and anti-inflammatory cytokines, albeit at lower concentrations, as well as angiogenic and profibrotic factors, including vascular endothelial growth factor (VEGF) and transforming growth factor (TGF)-*β* [83–88]. However, the role of mast cells in the FBR has been recently questioned when Yang et al. (2014) did not observe reduced or abrogated FBR markers in mast cell-deficient mice [89]. The role of T cells in the FBR has also been investigated when Rodriguez et al. (2009) demonstrated that, similarly, T cell-deficient mice were able to generate “normal” FBGCs and the FBR [90]. Both studies suggested the potential of compensatory mechanisms to account for their observations [91]. The involvement of T cells in the FBR to nonphagocytosable implants has not been fully elucidated; however T cells have been shown to attach to the biomaterial [92] and become activated through noncanonical pathways [93–96], as well as enhancing macrophage adhesion and fusion into FBGCs through paracrine actions of secreted cytokines [97–99].

The concerted action of immune cells results in pathways directed at isolating the biomaterial from the host tissue by fibrotic encapsulation [100] by the release profibrogenic factors such as platelet-derived growth factor (PDGF) [101–103], VEGF [104, 105], and TGF-*β* [106, 107] that recruit fibroblasts to the biomaterial (Figure 1(c)). Whilst the exact mechanisms of fibroblast recruitment have not been fully described, it has been suggested that biomaterial-adherent FBGCs serve as a constant source of fibrogenic mediators; however this remains to be tested [22]. Activated fibroblasts deposit collagen in an attempt to repair the damaged tissue; however excessive secretion results in fibrosis [108] and, in the case of implantation, forms a capsule around the biomaterial [109, 110]. In addition to collagen, other extracellular matrix (ECM) proteins act to scaffold and support tissue repair, thus presenting as network for signalling molecules and cell interactions [111, 112]. Following fibrotic encapsulation, the inflammatory responses may ultimately resolve if no infection is present; however the implant function may have been compromised by tissue repair, remodelling, and subsequent implant encapsulation processes [113, 114]. This well-described molecular and cellular iterative process strongly supports the notion that neutrophils and macrophage recruitment and response to biomaterial implant surfaces arbitrate the scope and magnitude of the subsequent foreign body response.

## 3. Innate Immunological Mechanisms Underpinning the FBR

The activation of leukocytes throughout the FBR is governed by controlled mechanisms that underpin innate immunity. Recognition and activation by leukocytes are dependent on surface receptor interactions that can be used to “sense” harmful situations. Evolutionary mechanisms that can detect foreign “stranger” pathogens are described by the interactions of pattern recognition receptors (PRRs) expressed on leukocytes with pathogen-associated molecular patterns (PAMPs) found on microorganisms [115]. These same PRRs can also recognise host “danger,” which may be induced by cell death, damage, or stress and can evoke immune responses. These responses are driven by molecules within the family of damage-associated molecular patterns (DAMPs). In the context of implantation, biomaterials are considered to induce “sterile” inflammation; therefore, the involvement of innate recognition and response mechanisms to DAMPs, which may be released by cells throughout the FBR, are described.

### 3.1. Stranger Danger: Outside the Cell Walls of Comfort

A subset of DAMPs are “alarmins” [116], which are endogenous molecules constitutively expressed and stored within intracellular compartments and, upon exposure to extracellular spaces, through passive release or active secretion, can evoke recruitment and activation of leukocytes [117]. Alarmins can be divided into eight categories, including cathelicidin, defensins, eosinophil-associated ribonucleases, heat shock proteins, ion-binding proteins, saposin-like granulysin, nucleotides/metabolites, and nucleotide-binding proteins [117]. Within the nucleotide-binding proteins category, high mobility group box 1 (HMGB1) is exemplarily as it fulfils all the requirements associated with an alarmin, which include (i) passive or active secretion following nonprogrammed or apoptotic cell death, respectively; (ii) production by immune cells without the need for dying; (iii) the ability to recruit and activate leukocytes; and (iv) involvement in reconstructing damaged tissue [118, 119]. Originally recognised for its role as a chromatin-associated protein involved in DNA transcription [120, 121], HMGB1 has since been described to present a role in danger sensing [122] and has since been associated with several pathologies including fibrotic diseases [123]. Extracellular HMGBI is a result of passive release from necrotic cells [124] or active secretion from leukocytes [125] and can act on a range of receptors, including receptor for advanced glycation end products (RAGE), which instigates its functionality [126]. In addition, HMGB1 has been reported as a chemoattractant for monocytes, macrophages, and dendritic cells (DCs) [127, 128] which is reliant on forming complexes with other cytokines [127]. The release of HMGB1 from platelets has also demonstrated the capacity to stimulate neutrophils as detected by ROS secretion [129]. A growing number of investigations have implicated a role for HMGB1 in fibrotic diseases, with increased levels of HMGB1 in patients that have systemic sclerosis [123, 130], cystic fibrosis [131, 132], liver fibrosis [133], or pulmonary fibrosis [134, 135]. In these settings, HMGB1 has been shown to affect fibroblast proliferation [136–138], migration [138, 139], and collagen synthesis [133, 137, 138] and enhance proinflammatory cytokine secretion [138, 140, 141]. Indeed, blocking HMBG1 with small interfering RNA in a murine model of liver fibrosis inhibited collagen production [133], whereas the injection of recombinant HMGB1 into mice could induce lung pathologies similar to that observed in cystic fibrosis [132]. There are limited reports investigating HMGB1 in biomaterial-related responses; however one study that used poly(lactic-co-glycolic acid) scaffolds as a model of subcutaneous biomaterial implantation could detect HMGB1 at the site of scaffold implantation but not in animals that underwent surgery without receiving the scaffold. This suggested that HMGB1 may be released by necrotic cells or lymphocytes due to tissue damage at the implantation site [142]. Ongoing investigation of extracellular HMBG1 in biomaterial-induced inflammation could be of considerable value as several methods of therapeutic or prophylactic HMGB1 blockade/inhibition have already been established in various* in vivo* models, which, with further exploration, may also be advantageous in situations of surgical biomaterial implantation where risks of tissue damage are high.

An additional nucleotide-binding alarmin, IL-33, may also be of interest for biomaterial investigations [117]. In a similar manner to HMGB1, IL-33 can act as both a cytokine and a nuclear factor and has been linked to fibrosis through the actions of leukocyte recruitment and modulation of ECM genes [143, 144]. Expression of IL-33 has also been shown to be increased in patients with idiopathic pulmonary fibrosis and liver fibrosis [145–147]. In murine models, overexpression of IL-33 in hepatocytes caused an excessive local immune cell infiltration and increased hepatic collagen deposition. This was however abrogated in IL-33^−/−^ mice, suggesting that IL-33 has a role in driving ECM deposition [148]. Furthermore, systemic administration of IL-33 to mice resulted in increased mRNA levels of IL-13 and the development of skin fibrosis [143]. In a manner relevant to its alarmin function, IL-33 is constitutively expressed and is released during necrosis as an active protein [145]. If IL-33 release is accompanied by the presence of neutrophils at a local injury site, serine proteases, cathepsin G, and elastase can cleave IL-33 to generate a “superactive” form of the protein [149, 150]. Upon secretion, IL-33 has been found to be a chemoattractant for Th2 cells [151], as well as acting directly on Th2 cells via constitutively expressed (protein of growth stimulation gene 2) ST2 receptor [152, 153] to induce the secretion of IL-13 [153, 154], an important cytokine detailed in FBGC formation. There are currently no studies that directly assess the involvement of IL-33 in biomaterial-induced inflammation; however the emerging links between IL-33 and fibrotic disorders might suggest that release of IL-33 at the biomaterial implantation site by necrotic cells may encourage the cytokine milieu that results in FBGC release and collagen deposition. Further assessment may qualify IL-33 as an additional candidate for therapeutic blockade to reduce the FBR.

### 3.2. Integrin Receptors

Traditionally viewed as adhesion receptors, integrin receptors have demonstrated their capacity to independently recognise DAMPs to induce immune responses. There are 22 integrin receptors in mammals based on the combinations of different *α* and *β* chain subtypes (17 *α* subunits and 8 *β* subunits) which form noncovalent heterodimers [155, 156]. The observation that integrin receptors can mediate proinflammatory cytokine release upon activation by bacterial components [157–159] was instigated by a study in 2005 that reported on how integrin ligands could prompt recognition by leukocytes [160]. Vorup-Jensen and colleagues demonstrated that the integrins *α*_2_ *β*_2_ (CD11c/CD18) and *α*_M_ *β*_2_ (Mac-1; CD11b/CD18) could recognise acidic residues exposed on proteins that were degraded by the action of bacteria-derived proteases [160]. The significance of this study was that misfolded or degraded proteins could be recognised by leukocyte-expressing receptors, which is not only engendered by bacterial challenges, but present in ECM remodelling processes [160]. In particular, fibrinogen is commonly investigated because it can bind several integrins through its arginine-glycine-aspartic acid (RGD) sequence [161], which is also found in other integrin ligands [155, 162] such as fibronectin [163] and vitronectin [164]. Interestingly, fibrinogen absorbed onto the biomaterial may alter its conformation due to chemical and physical properties, exposing RGD for leukocyte recognition via Mac-1, and initiate integrin signalling [165–167]. Therefore, the susceptibility of fibrinogen to lose structural integrity and bind leukocyte receptors may suggest that fibrinogen acts as a sentinel of tissue damage [160].

### 3.3. Toll-Like Receptors

Pattern recognition receptors are traditionally divided into five main families: toll-like receptors (TLRs), nucleotide-binding oligomerization domain- (NOD-) like receptors (NLRs), absent in melanoma- (AIM-) like receptors (ALRs), retinoic acid-inducible gene I- (RIG-I-) like receptors (RLRs), and the C-type lectin receptors (CLRs) [115, 168]. For the purpose of this review, TLRs, NLRs, and ALRs in the context of biomaterial-induced responses will be discussed.

There are 10 TLRs in humans [169, 170] and 12 TLRs in mice [169, 171], but both present as transmembrane receptors found on the plasma membrane and endosome/lysosome membranes [115] for PAMP and DAMP detection. Toll-like receptors are characterised by an extracellular leucine-rich repeat (LRR) and an intracellular toll/IL-1 receptor (TIR) domain [172]. Upon recognition of their distinguished ligands (reviewed elsewhere [173]), TLR signalling pathways can induce the secretion of proinflammatory cytokines or type one interferons, as mediated by the myeloid differentiation primary response gene 88 (MyD88) or TIR-domain-containing adapter-inducing interferon-*β* (TRIF) adaptor proteins, respectively [174].

Despite the predominant investigation of TLRs in inflammation and infection, reports of TLR involvement in the context of biomaterials are now being described, mostly for phagocytosable particles, including implant aseptic and septic loosening due to wear debris. It has been extensively demonstrated that TLR2 and TLR4 play a role in recognising the subclinical levels of bacterial contamination that drive implant loosening [175–178]; however it has also been shown that* in vivo* oxidised alkane polymers can directly induce TLR1/2 signalling [179]. In a subsequent* in vitro* model, oxidised alkanes were shown to have 140 times greater binding affinity to soluble TLR2 than the nonoxidised polymer [179]. These results begin to detail the innate response following the degradation of common materials used for implants; however further investigations are required to assess TLR interaction with nonphagocytosable biomaterials. With a focus on DC functionality, Shokouhi et al. (2010) used MyD88-deficient DCs for an assessment of global TLR actions in biomaterial responses [180]. The results of the study demonstrated that in comparison to wild-type DCs, MyD88-deficient DCs had lower surface marker expression and decreased cytokine secretion when they were incubated on a range of biomaterials. Individual TLR-deficient DCs were also subject to the same analysis and it was revealed that TLR1, TLR2, TLR4, and TLR6 had a role in recognising and responding to biomaterials as DC functionality was abolished or strongly impaired in these cells compared to wild-type DCs [180]. The importance of TLR4 was also observed by Rogers and Babensee (2010) in a study highlighting the effect of TLR4 on leukocyte recruitment, adhesion, and fibrotic encapsulation [181]. Interestingly, the results of this study did not define a role for TLR4 in the recruitment of leukocytes, the TNF*α* levels in peritoneal exudates, or the thickness of the fibrotic capsule. Instead, TLR4 deficiency seemed to impart differences in the profiles of adherent leukocytes on the biomaterial surface. The TLR4-deficient mice appeared to have increased neutrophils and decreased monocytes/macrophages adhered onto the biomaterial, in comparison to the TLR4-sufficient control mice [181].

In 2004, Seong and Matzinger proposed the hydrophobicity model to explain mechanisms of alarmin recognition by TLRs [182], an idea which may be plausible for the results observed in biomaterial models. The hydrophobicity model [183] stems from the understanding that hydrophobic portions or “hyppos” of molecules are usually hidden from the aqueous environment by conformational folding that result in functional aggregates. However, in situations when hyppos are exposed, specifically in damaged tissue, these regions act as universal signals of homeostatic disruption due to protein misfolding and potentially toxic and nonproductive aggregates, which are recognised by TLRs [182]. To date, this serves as a good model for justifying TLR involvement in biomaterial responses in sterile inflammation. Additionally, the hydrophobicity model may explain the fact why fibrinogen can act as an alarmin and has been shown to be a TLR4 ligand, presumably through the exposure of the RGD domain [184–188]. Other ECM proteins associated with biomaterial adsorption have also been reported as TLR ligands [189], including fibronectin [187, 190], as well as proteins associated with damage such as HMGB1 [191, 192]. The significance of understanding the roles of TLRs in FBRs was recently demonstrated in a study assessing the therapeutic efficacy of a TLR2/6 agonist. In this study, porous polyethylene (commercially known as Medpor) is used for craniofacial reconstructive surgery; however, adequate tissue integration relies on rapid vascularisation [193]. To this end, macrophage-activating lipopeptide-2, a TLR2/6 agonist, was locally injected into preclinical models investigating Medpor. The treatment was shown to increase vascularisation 14 days after implantation and did not cause local or systemic side effects as determined by control animals [193]. This study highlights the fact that targeting specific TLR pathways can force a desired response and therefore manipulating TLR signalling could potentially be used to control the FBR [194].

### 3.4. Cytosolic Sensors and the Associated Inflammasome That Control the Release of Potent Proinflammatory Cytokines

Whilst the TLR family can detect extracellular signals, the NLRs are soluble proteins located in the cytosol for intracellular monitoring of a broad repertoire of PAMPs and DAMPs [195]. Structurally, NLRs are composed of three domains: (i) the C-terminal domain containing LRRs involved in ligand sensing, (ii) a central nucleotide domain, NACHT domain (also referred to as NOD), responsible for oligomerization of NLRs, and (iii) the N-terminal effector domain that differs based on its exclusive composition of a caspase activation and recruitment domain (CARD), a pyrin domain (PYD), or a baculovirus inhibitor of apoptosis repeat (BIR) domain [196, 197]. The structural and functional diversity of domain composition classifies NLRs in three subfamilies: (i) NLRPs (also referred to as NALPs) based on PYD as the N-terminal, (ii) NODs which predominantly express CARD (also referred to as NLRCs), and (iii) the IPAF/NAIP (IL-1*β*-converting enzyme protease-activating factor/NLR family, apoptosis inhibitory protein) family whereby IPAF (synonymous with NLRC4) contains a CARD, and NAIF contains BIR domains (also referred to as NLRBs) [196]. Together, these cytosolic sensors can detect ligands from invading sources and initiate signalling pathways that result in the secretion in cytokines, interferons, and microbicidal proteins.

**Figure 2:**
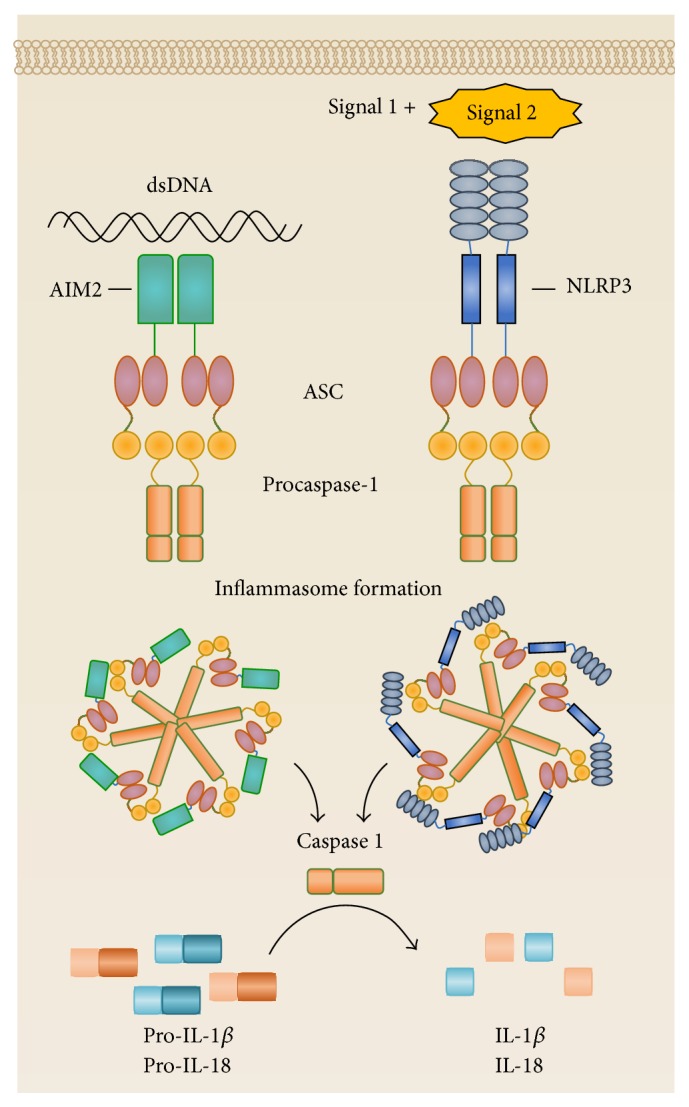
Activation of the inflammasome results in the secretion of IL-1*β* and IL-18. Inflammasomes are activated when sensors proteins detect stimulatory ligands. The AIM2 sensor binds double stranded DNA via the HIN200 domain. The NLRP3 sensor requires two signals for activation, instigated through TLR activation (signal 1) and followed by an indicator of homeostatic disruption (signal 2). Inflammasome formation is based on homotypic interactions of the components, whereby AIM2 and NLRP3 sensors proteins recruit ASC through their PYD, allowing ASC interactions with procaspase-1 via CARD-CARD associations. This multiprotein complex forms a spherical “wheel” structure to encourage proteolytic cleavage of procaspase-1 into caspase 1. Caspase 1 functions to cleave pro-IL-1*β* and pro-IL-18 into their active IL-1*β* and IL-18 forms, respectively.

A unique feature of some PRRs, including NLRs, is the ability to form an inflammasome. The term “inflammasome” was coined by Martinon et al. (2002) to describe high molecular weight complexes due to multiprotein assembly that activate inflammatory caspases and result in IL-1*β* secretion (Figure 2) [198]. The pioneering work of the Tschopp laboratory has led to the identification of seven inflammasomes, each named after their protein scaffold, including the NLRP1, NLRP3, NLRP6, NLRP12, IPAF inflammasomes, and, more recently, the IFI16 and AIM2 inflammasomes [197, 198]. The AIM2 and IFI16 proteins are two of eight cytosolic sensors in mice that comprise the ALR family [199], all of which contain a HIN200 (hematopoietic interferon-inducible nuclear antigens with a 200-amino acid repeat) domain at the C-terminal and can directly bind double stranded DNA (dsDNA) from various sources [200]. In particular, the AIM2 protein is not as structurally diverse as NLRs, as the C-terminal HIN200 domain is complemented with an N-terminal PYD [201]. The formation of canonical inflammasomes, regardless of sensor protein, relies on homotypic interactions between equivalent domains on target proteins. Therefore, upon activation of the cytosolic sensors, inflammasome assembly is initiated by the recruitment and binding of ASC (apoptosis-associated speck-like protein containing a caspase recruitment domain, also referred to as PYCARD) via PYD-PYD interactions [200, 202]. The C-terminal CARD of ASC facilitates interactions with the CARD of procaspase-1, resulting in its self-activation into caspase 1 by proteolytic cleavage into the active heterodimer comprised of 10 and 10 kDa subunits (p10 and p20, resp.) [203]. Together, the interactions between the cytosolic sensors, the ASC adaptor protein, and caspase 1 form the inflammasome (Figure 2). Structurally, inflammasomes may resemble the “apoptosome” required for caspase 9 activation based on the potential to form a double-ringed “wheel” structure of the multiple heterotetramers complexes [204, 205]. The central aggregation of caspase 1 is thought to lock caspase 1 into an enzymatically active state to facilitate the cleavage of pro-IL-1*β* and pro-IL-18 into their biologically active forms, IL-1*β*, and IL-18, respectively (Figure 2) [206].

Another outcome of inflammasome activation is a form of nonhomeostatic and lytic mode of cell death, termed pyroptosis, which requires the activity of caspase 1 or caspase 11 depending on the stimuli [204]. The activation of caspase 11 has been implicated in inflammasome activation and, however, is unable to process pro-IL-1*β* and pro-IL-18 and directly influences inflammasome-mediated pyroptosis [204, 207]. Interestingly, cytokine production precedes the induction of pyroptosis, suggesting that the cell inflicts maximal inflammation to promote immune activation prior to its death. It is thought that pyroptosis serves to prevent intracellular pathogen replication by eliminating the infected cell and also enhancing pathogenic recognition by exposing the pathogen to circulating neutrophils and phagocytes [204]. Consequently, cells that have undergone pyroptosis may also release endogenous molecules into the extracellular milieu as danger signals, of which HMGB1 and IL-1*α* have shown to be passively secreted [208–211], further perpetuating the inflammatory response. Considering the potent nature of IL-1*β* and IL-18, it is understandable that inflammasomes require regulators to control activation and efficiently subside inflammatory signals. The two major types of inflammasome regulators are proteins that contain either a CARD to prevent ASC recruitment or PYD-containing proteins to disrupt sensor-ASC interactions [212, 213]. Together, PRRs and inflammasomes present an impressive and controlled mechanism for alerting the host of stranger and danger signals.

### 3.5. Activating the NLRP3 and AIM2 Inflammasomes

Traditionally, inflammasomes are concerned with the control of invading pathogens such as bacteria [214, 215], viruses [216, 217], or fungi [218, 219] by secreting IL-1*β*, which is a potent proinflammatory cytokine. It is recognised that NLRP3 inflammasome activation requires two signals. The first signal is a “priming” step and results in the production of pro-IL-1*β* via transcription factor NF*κ*B-mediated regulation, commonly achieved by TLR stimulation [220]. Interestingly, TLR-induced NF*κ*B activation has also been implicated in the transcriptional control of NLRP3 and can increase NLRP3 expression to potentiate intracellular sensing [221, 222]. Unlike pro-IL-1*β* transcription, pro-IL-18 is constitutively expressed in macrophages and does not rely on NF*κ*B-mediated transcription [223, 224]. The second signal required for NLRP3 activation consists of a broad range of infection or stress-associated signals, and due to the diverse nature of these signals, it is unlikely that direct interactions with NLRP3 would induce its activation [197]. Instead, NLRP3 activation has been implicated to occur through the induction by three main methods: (i) ion flux, (ii) ROS, and (iii) lysosome rupture [197]. In the ion flux method, changes to cytosol concentrations of hydrogen (H^+^), calcium (Ca^2+^), or potassium (K^+^) ions disrupt intracellular homeostasis and activate NLRP3. In particular, extracellular ATP released from damaged or stressed cells can act on the P2X7 ion channel to trigger K^+^ efflux [225–227]. Endothelial and epithelial cells have been shown to release ATP in situations of mechanically induced stress such as compression [228], stretching [229], and changes in blood flow [230] and may result in K^+^ efflux [196]. Similarly, H^+^ efflux or toxic levels of cytosolic Ca^2+^ can activate the NLRP3 inflammasome [220, 231, 232].

The second method is defined by the release of ROS as an indicator of oxidative stress, which is induced by many NLRP3 stimuli such as ATP, alum, uric acid, or nigericin [226, 233, 234]. However the role of ROS in NLRP3 activation remains controversial. Contradicting reports suggest that ROS solely acts to upregulate NLRP3 and pro-IL-1*β* expression in an NF*κ*B-dependent manner [235], and other studies demonstrated how ROS can be sensed by a complex of Thioredoxin (TXN) and TXN interacting protein (TXNIP) which binds to NLPR in conditions of oxidative stress [236]. Recently, Shimada et al. (2012) proposed a unified model of NLRP3 activation and suggested that, in the presence of signal one, the NLRP3 stimuli can cause mitochondrial dysfunction, resulting in apoptosis and NLRP3 activation [237]. This study also demonstrated that apoptosis results in the release of ROS and mitochondrial DNA and that oxidised mitochondrial DNA could directly interact with NLRP3 as observed by immunoprecipitation [237].

The third method for NLRP3 activation is based on the detection of lysosome rupture during frustrated phagocytosis caused by large particulates, including uric acid crystals, alum, silica, and asbestos [233, 238]. It was demonstrated that frustrated phagocytosis could alter the cytoskeleton, and upon disruption of the actin filaments with cytochalasin D, the secretion of IL-1*β* was inhibited [238]. Furthermore, NLRP3 activation can be subsided with the treatment of cathepsin B, a lysosomal protease inhibitor [233]. Despite the understanding of downstream responses of phagocytosis of particulates, the exact molecular links that connect these events to NLRP3 activation remain to be elucidated [233].

Originally documented as a cytosolic HIN200 family protein for binding dsDNA, AIM2 was recently identified as an inflammasome-inducing sensor that could aggregate with caspase 1 and ASC [200]. The activation mechanisms of the AIM2 inflammasomes have not been detailed to the same extent as NLRP3 but do not appear to rely on a two-hit signal model, most likely due to the unambiguous HIN200 domain directly binding foreign dsDNA from viruses, bacteria, or self-dsDNA from cells that have undergone apoptosis [216, 239]. Upon DNA binding, AIM2 undergoes a conformational change and oligomerises around the DNA [240], recruiting ASC and caspase 1 for inflammasome formation, thus IL-1*β* and IL-18 secretion, and pyroptosis [200, 241, 242]. Recently, mitochondrial contributions to AIM2 inflammasomes were implicated as mitochondrial ROS was shown to potentiate AIM2 inflammasome activation in response to bacterial challenge [243] and mitochondrial DNA could directly bind AIM2 in its oxidised and nonoxidised form [237]. Interestingly, HMBG1 in complex with DNA was shown to induce AIM2 inflammasome activation, but upon induction of autophagy, HMGB1-DNA complexes were no longer able to trigger IL-1*β* release, suggesting an autophagy-mediated negative feedback pathway [244].

### 3.6. The Role of Inflammasomes in Biomaterial-Induced Inflammation

The observation that phagocytosable particles, such as asbestos and silica, could activate NLRP3 inflammasomes was investigated to understand the fibrotic diseases they caused [238]. Asbestos is an insulating material that was widely used in construction around the 1970s and was later discovered to cause several lung-related diseases including mesothelioma, lung cancer, and asbestosis [245]. Crystalline silica (silicon dioxide) is the most abundant mineral worldwide and is commonly encountered in areas of mining, construction, and farming. Inhalation of silica is associated with numerous pathologies including tuberculosis, lung cancer, chronic obstructive pulmonary disease, and silicosis [246]. The observation that alveolar macrophages could secrete IL-1*β* upon asbestos or silica exposure naturally led to the discovery that NLRP3 was involved in their related pathologies [238, 247]. The mechanisms of NLRP3 activation by asbestos and silica particles are similar to those described for invading pathogens. Both asbestos and silica can induce the generation of ROS [247–249] and, in the case of asbestos, works to indirectly activate NLRP3 by irreversibly oxidising TXN, resulting in the dissociation of TXN-TXNIP and subsequent binding of TXNIP with NLRP3 [250]. Thus, the roles of asbestos and silica seem to be indirect, whereby particle phagocytosis results in lysosomal swelling and damage, allowing NLRP3 to “sense” this perturbation [233]. The activation of the NLRP3 inflammasome has been implicated in driving inflammatory responses to nanoparticular carbon [251] and polystyrene [252], as well as nanodebris typically derived from implants [253] including amorphous silica and titanium dioxide (TiO_2_) [254], CoCrMo [255], and silver [256] (reviewed in [257]). However, it is unclear whether the NLRP3 activation mechanisms induced by these materials are consistent amongst the various phagocytosable particles.

The involvement of the inflammasome has also been implicated for large biomaterials that cannot be phagocytosed or do not generate wear debris or particulates. Detection of IL-1*β* at the local implant site [258] and* in vitro* quantification of IL-1*β* secretion by biomaterial-adherent macrophages [259, 260] suggest, at least in part, a role for the inflammasome in biomaterial-induced inflammation, although inflammasome-independent pathways of IL-1*β* secretion have recently been described [261–263]. The proposed models of NLRP3 inflammasome activation also overlap with certain events described within the FBR, such as ROS production by neutrophils and frustrated phagocytosis by macrophages. The associated tissue damage of implantation may also result in ATP release through mechanically induced cell stress leading to K^+^ efflux; however this has not been directly shown in the FBR and further investigation is required to decipher if there are any correlative relationships between these events and NLRP3 inflammasome activation. Malik et al. (2011) were the first to demonstrate the direct involvement of ASC and caspase 1 in the progression of FBR events, with mice deficient in these components exhibiting thinner fibrotic capsules around silicone disk implants at 4 weeks after implantation [264]. Interestingly, the absence of NLRP3 or NLRC4 sensors did not affect capsule thickness compared to controls, and this may suggest the involvement of another ASC-dependent inflammasome or, alternatively, inflammasome activation-independent mechanisms [215, 265, 266] that can indirectly affect the FBR. However, when assessing “acute” stages of FBR with the injection of PMMA beads of ~153 *μ*m diameters, NLRP3, ASC, and caspase 1 were involved in leukocyte recruitment within 24 hrs [264]. The results of this study also supported a recently described method of inflammasome activation, independent of phagosomal disruption. It was shown that interactions of solid structures with the cholesterol regions of lipid rafts of the cell membrane could activate the Syk kinase [267]. Malik et al. (2011) showed a reduction in IL-1*β* secretion when macrophages were pretreated with a cholesterol-depletion agent or a Syk inhibitor, supporting a membrane affinity triggered signalling (MATS) mode of inflammasome activation [264]. This was an extension from a study that investigated how DC activation could be induced via surface contact with particulates, namely, MSU crystals. The authors proposed a two-step process: firstly the MSU crystal lattice is aligned with cholesterol in the DC membrane to form tight interactions within 30 seconds of contact, which leads to lipid rearrangement. Secondly this lipid sorting results in the aggregation of immunoreceptor tyrosine-based activation motif- (ITAM-) containing receptors into cholesterol/sphingolipid rich regions of lipid rafts and initiates Syk kinase pathways [267]. Therefore, understanding the mechanisms of potential inflammasome components that are activated by large biomaterials and contribute to the FBR may result in clinically translatable methods of reducing inflammatory outcomes, such as kinase inhibitors [268] or the IL-1 receptor (IL-1R) antagonist, anakinra [269].

A current gap in the literature is the role of the AIM2 inflammasome in sterile environments, particularly in biomaterial-induced inflammation. Further investigation of AIM2 in these pathologies is not unwarranted on the basis that AIM2 can recognise self-dsDNA as a product of DAMP-induced cell death and may affirm an indirect role of alarmins. We have recently begun exploring the role of AIM2 in biomaterial-induced inflammation by adapting the model of PMMA bead injection [264] to induce pathways of the FBR in a murine model. Results in AIM2^−/−^ mice demonstrated a reduced capacity of neutrophils and macrophages to migrate to the injection site, as well as early collagen deposition when compared to B6 control mice (Christo et al., manuscript in preparation). The implications of this study are still under investigation but importantly highlight that, in the absence of the AIM2 sensor, the innate immune responses are skewed; however whether this is inflammasome activation dependent or independent remains to be determined.

## 4. Shaping and Tuning Innate Immune Effector Responses to Biomaterial Implants

The modification and derivatization of implant surfaces are being investigated to improve the biocompatibility of biomaterials. The ability to “mimick” the biology of the surrounding tissue or promote wound repair is thought to improve the integration of biomaterials and thus reduce the FBR [28, 270–272]. Despite successful attempts to redirect innate responses to constructive healing processes, materials such as alginate, collagen, intact, and decellularized ECM and ECM components are also susceptible to normal destructive tissue remodelling and unwanted metabolic breakdown products. Reproducing these materials may also be unfavourable for regulatory and manufacturing processes, and therefore synthetic polymers have been explored for surface coatings. One example of this is hydrogel coatings (PHEMA, PEG, and PVA), which become swollen with water, are highly permeable to analytes, and have mechanical properties akin to soft tissue. However, hydrogels and other synthetic polymers are limited in their adhesive abilities onto biomaterial scaffolds and, for this reason, are able to induce the FBR and, in some cases, are more likely to be degraded via macrophage and FBGC-mediated responses, ultimately leading to device failure. Therefore, two heavily investigated biomaterial modifications are alterations in surface chemistry and topography.

### 4.1. Surface Chemistry

The understanding that surface function alters protein adhesion and directs downstream cell recruitment has led to the global aim of producing “repellent” surfaces to moderate protein binding [273]. Methods of altering surface chemistry are vast but may be achieved by chemical grafting, self-assembled monolayers, or plasma polymerisation, and each has been tested for ultimately controlling the amount and composition of protein adhesion as well as conformational changes of the bound protein. It is well accepted that proteins will bind more strongly to hydrophobic surfaces compared to hydrophilic surfaces; however poor translation of these observations into* in vivo* outcomes have encouraged revised investigation into “ideal” properties such as, functional groups and wettability, and surface charge.

The most commonly explored functional groups are amino (–NH_2_), carboxyl (–COOH), hydroxyl (–OH), and methyl (–CH_3_) groups [273]. Of the hydrophilic surfaces, –NH_2_ and –OH groups present positive and neutral charges, respectively, and have been reported to induce the highest infiltration of inflammatory cells* in vivo* [274–276] and thicker fibrotic capsules around the functionalised implant [276, 277]. However, in a study by Barbosa et al. (2006), implants functionalised with hydrophobic, neutral –CH_3_ groups formed thicker capsules than –OH groups and also recruited higher numbers of Mac-1^+^ cells [278]. The authors suggested that, in their model, there was a directly proportional relationship between the severity of the acute inflammatory response and the thickness of the fibrotic capsule [278]. The implications of this simplistic model may not be translatable to all systems, and, in fact, there are contradictory reports on the ability of –CH_3_ groups to promote cellular adhesion [274, 279, 280]. It is therefore important to recognise differences between* in vitro* and* in vivo* studies, which may account for this disparity.

In the context of cell differentiation pathways, surfaces with –OH functionality show the highest capacity to induce osteoblast differentiation, followed by –NH_2_ and –COOH, and the lowest capacity on –CH_3_ surfaces [281, 282], although –CH_3_ surfaces seemed to induce moderate myogenic differentiation [281]. Blocking antibodies revealed that cell differentiation was regulated by integrin binding [283] in alignment with observations of preferential focal adhesions formation on –OH > –NH_2_ = –COOH > –CH_3_ surfaces [282]. Focal adhesions are specialised regions of the cell that are rich in integrins and signalling molecules that allow attachment to the ECM and serve as signalling centres to regulate cell growth, survival, and gene expression [284]. Further evidence of focal adhesion dependence on surface chemistry was seen on –NH_2_ surfaces that could regulate the expression of integrins and signalling molecules resulting in ERK1/2 activation in bone marrow stromal cells, which have osteogenic differentiation ability [285].

Not surprisingly, the differential involvement of integrin engagement coincides with the understanding of how adsorbed proteins can alter their conformation. Protein studies revealed that the ability to access fibronectin domains, integrin binding, and cell adhesion followed the order of –OH > –COOH = –NH_2_ > –CH_3_ surfaces [286, 287]. Interestingly, the complement component C3b can covalently link to OH [288, 289] and its inactive form, C3bi, is the receptor for Mac-1 [290], potentially offering an explanation for increased accumulation of CD11b^+^ cells (macrophages and neutrophils) at the implant site [291]. Interestingly, –COOH is recognised for imparting minimal inflammatory damage based on the observations that these hydrophilic and negatively charged surfaces have consistently thinner fibrotic capsules and reduce cell infiltrates at the implantation site [276, 278, 291]. Whilst repulsion of negatively charged cell membranes may account for some of these observations, the role of protein binding on these surfaces offers a more cohesive explanation. It has been found that hydrophobic surfaces including –CH_3_ can more tightly bind proteins such as albumin [292, 293] and fibrinogen [293, 294] and cause conformational changes [292], whereas hydrophilic surfaces show faster protein desorption [295]. However, simple distinction of hydrophilicity is not sufficient to predict the protein binding kinetics of a given surface and, instead, a second tier of differentiation by way of surface charge is required. Sivaraman et al. (2009) showed that proteins on –OH groups were able to retain their native structure to a much greater extent than any other functional group including the equivalently hydrophilic –COOH, which differs on the basis of its negative charge [292]. The authors suggested that the internal hydrophobic regions of a given protein are less likely to unfold in the presence of a hydrophilic surface and instead form hydrogen bonds with –OH groups via interactions of polar and charged amino acid residues presented on the protein surface. Interestingly, the observation that proteins adsorbed to –COOH surfaces showed greater structural rearrangement than their native forms was attributed to the negatively charged –COOH groups interacting with positively charged amino acid residues on the protein, perturbing the lowest free energy state of that protein, causing it to refold to the new lowest free energy state conformation. Similar observations and rationale were described for proteins on the hydrophilic and positively charged –NH_2_ surfaces, resulting in greater conformational changes [292]. Together, protein binding kinetics and conformations on implant surfaces are dependent on surface chemistry and may contribute to explaining the FBR results* in vivo*.

### 4.2. Surface Topography

Methods of altering topography are wide and varied, including, but not limited to, particle deposition, self-assembled monolayers, soft photolithography, blasting, acid etching, and polymer expansion [296–298]. These techniques result in differential surface geometries in the micron and nanometre scale, producing “rough” surfaces based on protrusions, such as pillars, posts, gratings, and ridges, or dentations, such as pits and dots [104, 299–301]. Comparison of native substrates with modified topography is assessed by the functionality of cells that are of interest to the downstream application of the material, which may include osteoblasts in the case of bone implants but are mostly concerned with cells involved in the FBR. Interestingly, the notion of modulating surface “roughness” using micro and nanopatterns stems from the architectural understanding of homeostatic cell interactions with components of the ECM [302]. The ECM is a complex structure of intertwined pores, fibres, ridges, and protein bands in the nanoscale [303, 304]. The topographical control of cell interactions has inspired the investigation of synthetically produced disturbances for modulating implantation outcomes [302]. In general, “rougher” surfaces have shown altered cell adhesion [305–307], density and spreading [308, 309], modulated cytokine secretion [310, 311], motility [312, 313], enhanced proliferation and differentiation [308, 314], and macrophage fusion [104]; however these responses are cell-specific and dependent on the method of fabrication.

Additionally, surface roughness is further differentiated based on modified dimensions such as topography height, width, rigidity, and spacing and patterns. For example, Mohiuddin et al. (2012) used nanodots of various diameters to show that macrophage secretion of IL-6 was doubled on 50 nm nanodots compared to smooth surfaces but increased 3-fold on 200 nm nanodots. Interestingly, maximum cell spreading, focal adhesion, and cell density were seen on the 50 nm nanodots [301]. In a similar manner, Hulander et al. (2013) immobilised nanoparticles of different sizes and demonstrated that 56 nm particles decreased platelet activation; however the 36 nm particles allowed platelet contact with the “flattened” surface, inducing activation and cell flattening [306]. On the micron scale, Madden et al. (2010) revealed differences with pillars of different distances, where larger gratings, that is, 1 *μ*m versus 250 nm distances, reduced macrophage fusion and cytokine secretion. Recently, differences in macrophage phenotype were shown to be regulated on surfaces anodised with 5 V or 20 V, where surfaces generated with 5 V caused macrophages to secret cytokines of an “M2” phenotype, whereas the rougher 20 V surfaces were associated with an M1 phenotype. Similar analysis of macrophage polarity has been described for porous versus nonporous poly(2-hydroxyethyl methacrylate-co-methacrylic acid) hydrogels showing that when compared to the nonporous hydrogels, implanted hydrogels with pores of 30–40 *μ*m have greater neovascularisation and reduced fibrotic capsule [315]. Furthermore, porous hydrogels had a significant increase in the proportion of macrophages expressing macrophage mannose receptor (MMR) and a significant decrease in MMR^−^ cells, suggesting a phenotypic shift into an M2 phenotype [315]. These reports in conjugation with studies assessing macrophage polarity in the context of other scaffolds highlight the importance of a deeper analytical approach to immunophenotyping infiltrates and resolving proportional contributions of these cells towards signalling pathways and cytokine responses. Macrophage responses were assessed in a study by Bota et al. (2010) that used expanded polymers to create pores with different intranodal distances. The authors found that the largest distance of 4.4 *μ*m produced the highest level of macrophage-derived IL-1*β in vitro* but, importantly, resulted in a thinner capsule surrounding implant* in vivo* [299]. The use of porous materials has been investigated for several decades and has been integrated into areas of orthopaedic implants, such as dental and bone (joint) implants. In general, the porous nature of these implants is ideal because they allow for tissue integration, vascularisation, and the transport of nutrients [316, 317]; however host responses can vary based on the scaffold material, pore size, and porosity. Although the ideal pore size for osteoblast functionality in implants for bone engineering is still disputed [318], pores ranging within 20–1500 *μ*m [319] have been investigated for cell migration, proliferation, osteogenesis, and angiogenesis [320–322]. Importantly, the porosity and pore size must also be considered from the perspective of the implant's mechanical properties to ensure that its strength is not comprised [318]. Collectively, disparity amongst various surface topographies highlights the importance of using cell types specific to a given implant function in order to assess the optimal properties that result in the desired response* in vivo*.

However, cellular responses are secondary to protein adsorption in an* in vivo* setting, and, for this reason, the effects of nanotopography on protein binding have also been investigated. Surface roughness was shown to increase serum protein adsorption by 70%; however inspection of specific proteins on these gold nanoparticle (58 nm) surfaces revealed that the increase in protein was due to IgG antibody binding, whereas complement C3c decreased by 50% [323]. Further investigations have detailed the effect of nanotopography on altered adsorption and conformation of fibrinogen [324, 325] and fibronectin [326, 327], potentially accounting for differences in cell adhesion and activation on flat versus rough surfaces. It is suggested that alterations in protein binding are due to surface “curvature,” which can affect two main factors: (i) the orientation, unfolding or distances between adsorbed proteins, and (ii) distortion of the cytoskeleton upon membrane conformation on the shape of the curve [302, 328]. Together, these support the involvement of mechanotransduction as a mechanistic explanation for cell detection of surface topography. Mechanotransduction describes a process of how cells can relay a multitude of mechanical forces into the nucleus through the action of the cytoskeleton [329, 330]. It has been proposed that, in response to tension, cytoskeletal filaments reorient, causing the nucleus to distort and align against the axis of tension, potentially affecting nuclear scaffolds such as chromatin [331]. It has also been suggested that focal adhesions are the initiating site of mechanotransduction [332], offering an attractive rationale for topography-dependent cell response. In fact, several studies have shown surface-adherent cells can extend filopodia [333–335], which are finger-like protrusions of the cell membrane that are rich in actin and are found to contain integrins [336]. A study by Collie et al. (2011) used blocking antibodies to demonstrate a direct role for integrins in macrophages responses to topography, specifically showing that anti-*β*2 integrins abrogated IL-1*β* production, whereas blocking *β*1 *α*_v_ *β*_3_ integrins had no effect on macrophage responses [300].

The use of scanning electron microscopy has revealed unique cell behaviours on nanostructures [337] and provided insight into different forms of membrane contortion. Cells on patterned nanopillars have been shown to “bend” the pillars, most likely due to filopodia contraction; however, the ability to “tilt” these structures was dependent on the rigidity and stiffness of the material [338]. In some cases, cells attempted to phagocytose nanostructures, potentially utilising the increased surface area to promote greater points of contact for “grip” [308, 339]. Interestingly, it was recently observed that nanostructures could penetrate fibroblasts, which the authors suspected to be due to failed phagocytosis, resulting in cell thinning and membrane rupture [339]. Together, these studies highlight how the multitude of techniques and topography can differentially affect cell responses.

### 4.3. Modulating Inflammasome Components by Biomaterial Surface Properties

The goal of shaping innate effector immune cell responses within FBR pathways has been investigated in many different contexts from alternating biomaterial surface properties through to inhibiting innate effector cell outputs. However, how surface characteristics can manipulate innate effector cell signalling pathways requires further investigation. Currently, the MATS model is the most plausible hypothesis available. It has been demonstrated that membrane curvature as a result of cytoskeletal changes can reorganise local lipid composition and expose hydrophobic cavities [340–342]. This may in fact support the independent observations documented by Malik et al. (2011) that suggested a role of cholesterol and Syk in macrophage-derived IL-1*β* when in the presence of PMMA microspheres [264]. More recently, surface contact between macrophages and MSU crystals was shown to be sufficient to cause IL-1*β* secretion* in vitro* via the NLRP3 inflammasome. The authors demonstrated that the release of IL-1*β* upon MSU crystal contact was a result of K^+^ efflux, offering an additional mechanism that may result from membrane perturbations. It is known that K^+^ efflux is one model of NLRP3 activation and was recently found to be the common outcome amongst NLRP3 stimuli [343]. Therefore, the notion that surface receptor-independent signalling can be initiated upon changes to membrane curvature opens the possibility of assessing how surface topography can be used to modulate these responses. In this way, biomaterial-adherent cells may conform their membrane to the curvature of the surface topography, which may result in membrane lipid sorting and Syk kinase activation and the opening of ion channels to permit signalling, as well as inducing mechanosensing pathways, in a concerted manner that activates the innate effector cell.

The physicality of cell membrane conformations on various topographies is not to overlook the potential role of surface chemistry in controlling innate effector cell responses at a molecular level. Unfortunately, studies that assess the signalling pathways within biomaterial-adherent cells on various chemistries are minimal. A recent report comparing two zwitterionic hydrogels demonstrated that, when compared to poly(2-hydroxyethyl methacrylate) (PHEMA) hydrogels, polycarboxybetaine methacrylate (PCBMA) hydrogels induced less inflammation* in vivo*, thinner fibrotic capsule at 4 weeks and 3 months after implantation and supported vascularisation and more macrophages with anti-inflammatory phenotypes* in vivo* [344]. Importantly, this study demonstrated that changes to inflammatory responses were a result of the hydrogel chemistry and not due to endotoxin contamination, a fact that many other studies often neglect to mention or do not test for. The authors propose that PCBMA hydrogels were superior to PHEMA because they could resist protein adsorption to a greater extent, thereby reducing recognition by innate effector cells, namely, macrophages [344]. This then raises the argument that innate effector cell modulation could be achieved indirectly by controlling protein adsorption to eliminate receptor-mediated signalling. How this may in turn affect inflammasome activation is not yet determined and will require extensive investigations both* in vitro* and* in vivo*. There are many considerations to account for prior to translating preclinical therapeutic interventions into strategies for humans. A single approach may not adequately address clinical complications, and for this reason targeting different facets of the FBR may improve patient outcomes, whereby surface modification would alter protein adsorption and acute cell infiltration, allowing a more manageable inflammatory response for the administration of innate cell-targeted therapies.

## 5. Future Directions

As the field of biomaterials becomes infiltrated with the medical and biological sciences, it is evident that a deep and mechanistic understanding of immunological responses to implants is at the forefront of biocompatibility and tissue integration. In order to implement rationally designed implantable biomaterials, it is necessary to quantify the full breadth and temporal allocation of immune responses that contribute to downstream FBR events such as fibrotic encapsulation and biomaterial rejection [345]. Whilst* in vitro* methods are important for selecting materials and designs that may hold promise, the use of culture based techniques limits the full understanding of complex immune interactions. To this end the development of more robust and predictive* in vivo* models of biomaterial-induced inflammation is required for use with the appropriate genetically modified stains of mice deficient in key innate immune effector response pathways to ultimately assess the effect of physicochemical surface modifications on the initiation and progression of the FBR. It is important to gain a detailed understanding of the innate immune inflammatory processes by which neutrophils and monocytes/macrophages can be activated by biomaterial surfaces in the absence of any specific cell surface receptor or cytosolic receptor signalling. Furthermore, there is potential for exploring whether ASC-mediated inflammasome assembly is an important driver of the FBR and whether the surface nanotopography and the chemical reactive potential of an implant can modulate these responses. The relationship between how the physical characteristics of the surface of a biomaterial implant can affect inflammasome-dependent FBR initiation and progression will be mechanistically resolved using genetically modified mice made deficient in the key signalling pathways that are predicted to be the major arbiters in how this occurs and will also allow definition of the rules by which biomaterial surface nanotopography and chemical reactive potential change how the FBR is initiated and progresses.

## 6. Conclusions

The biggest challenge in the field of biomaterials is the understanding and, importantly, the prediction of long-term biological responses in patients receiving implantable biomaterials [29]. Deconstructing and detailing these mechanisms will allow for more targeted approaches and highlights how immune processes are amenable to manipulation by synthetic biomaterials. We anticipate that future explorations in this field of research will ultimately facilitate rationally designed and manufactured biomedical implants with substrate surface characteristics that will enhance utility, function, and clinical application.
